# The Association of Desert Dust with the Risk of Acute Coronary Syndrome in Subjects of a Younger Age

**DOI:** 10.3390/jcm13082392

**Published:** 2024-04-19

**Authors:** Alberto Domínguez-Rodríguez, Néstor Baéz-Ferrer, Pablo Avanzas, Sergio Rodríguez, Pedro Abreu-González, Elisa Trujillo-Martin, Guillermo Burillo-Putze, Daniel Hernández-Vaquero

**Affiliations:** 1 Departamento de Medicina Interna, Universidad de La Laguna, Servicio de Cardiología, Hospital Universitario de Canarias, 38320 Tenerife, Spain; 2 Centro de Investigación Biomédica en Red Enfermedades Cardiovasculares (CIBERCV), 28029 Madrid, Spain; avanzas@secardiologia.es; 3 Hospital Universitario de Canarias, Servicio de Cardiología, 38320 Tenerife, Spain; nestor.baez@hotmail.com; 4 Cardiology Area, Hospital Universitario Central de Asturias, 33011 Oviedo, Spain; 5 Instituto de Investigación Sanitaria del Principado de Asturias, 33011 Oviedo, Spain; dhvaquero@gmail.com; 6 Department of Medicine, Faculty of Medicine, Universidad de Oviedo, 33006 Oviedo, Spain; 7 Institute of Natural Products and Agrobiology (IPNA), CSIC, 38206 La Laguna, Spain; sergio.rodriguez@csic.es; 8 Unidad de Fisiología, Departamento de Ciencias Médicas Básicas, Universidad de la Laguna, 38200 Tenerife, Spain; pabreugonzalez@gmail.com; 9 Hospital Universitario de Canarias, Servicio de Reumatología, 38320 Tenerife, Spain; elisatm@telefonica.net; 10 Hospital Universitario de Canarias, Servicio de Urgencias, 38320 Tenerife, Spain; gburillo@telefonica.net; 11 Faculty of Health Sciences, Universidad Europea de Canarias, 38320 La Orotava, Spain; 12 Cardiac Surgery Department, Hospital Universitario Central de Asturias, 33011 Oviedo, Spain

**Keywords:** desert dust, acute coronary syndrome, premature coronary heart disease, air pollution

## Abstract

**Objectives:** Recently, desert dust in Europe has been recognized as a cardiovascular health problem. In Spain, desert dust inflows in recent years have been associated with worsening air quality. The present study examines whether desert dust events are related to the incidence of acute coronary syndrome (ACS) in patients under 55 years of age. **Methods:** Data from 2416 consecutive patients admitted to a tertiary hospital due to ACS were prospectively analyzed. A case-crossover time-stratified design using Poisson conditional regression models was applied to estimate the impact of desert dust events involving particulate matter concentrations of an aerodynamic diameter <10 μm (PM_10_) on the incidence of ACS in patients under 55 years of age. **Results:** Desert dust intrusion on days 0 to 5 before ACS onset showed no significant association with the incidence of ACS in patients under 55 years of age. The incidence rate ratios of PM_10_ concentrations 1, 2, 3, 3, 4, and 5 days before ACS onset (for changes of 10 µg/m^3^) were 1.02 (95% CI 0.97–1.1; *p* = 0.41), 1.01 (95% CI 0.96–1.07; *p* = 0.66), 0.99 (95% CI 0.94–1.05; *p* = 0.78), 0.96 (95% CI 0.9–1.02; *p* = 0.18), and 0.97 (95% CI 0.91–1.04; *p* = 0.41). **Conclusions:** Our findings suggest that desert dust is unlikely to be related to the incidence of ACS in patients under 55 years of age.

## 1. Introduction

The inhalation of combustion-related atmospheric pollutants has become a worldwide threat to human health. According to the World Health Organization (WHO), particulate material (PM) with an aerodynamic diameter of <10 μm (PM_10_) and <2.5 μm (PM_2.5_) has a very negative health impact [1]. The health consequences of the inhalation of desert dust particles carried by the wind in environmental air as a consequence of desert dust storms are becoming of growing interest throughout the world [1].

Northern Africa is the main world source of desert dust, representing 50–70% of the global emissions. The dust from northern Africa is regularly transported over the north Atlantic and also episodically over the Mediterranean and Europe [2]. Exposure to Saharan dust causes airway inflammation [3] and is associated with a 2% rise in cardiovascular mortality for every 10 µg/m^3^ increase in the concentration of PM_10_ [4].

The accelerated inflammation, vulnerability to plaque rupture, and favoring of thrombus development are key factors in the air-pollution-triggered occurrence and aggravation of an ACS [3]. Despite extensive investigations, it is unclear how Saharan dust events leading to high PM_10_ level can initiate an ACS. Particular air pollutants may enhance atherothrombotic processes via the generation of pulmonary inflammation or direct translocation into the systemic circulation, where PM may increase the risk of ACS [3]. Nevertheless, the exact pathogenic mechanisms by which the exposure of desert dust increases the risk of ACS still need to be fully elucidated.

Based on the age at disease onset, ACS is considered premature when it develops in young individuals (<55 years of age), similar to the definition of premature coronary artery disease [5]. The manifestation of ACS in young subjects is associated with an important decrease in life expectancy [5]. To our knowledge, no previous studies have related desert dust exposure to ACS in subjects of a younger age.

This study is a sub-analysis of a previous study of our group [6], and its aim was to explore the association between desert dust exposure and the incidence of ACS in patients under 55 years of age.

## 2. Methods

### 2.1. Study Population

The study was carried out in Tenerife (Canary Islands, Spain). The climate is characterized by trade winds (March to August) that contribute to rapid dispersal of the local atmospheric pollutants and help to preserve quite good air quality. Although desert dust episodes may occur over the course of the year, the two dust seasons are separated by the climate conditions: (a) November to March and (b) July to August [2].

The study was carried out in a tertiary hospital and included all patients diagnosed with ACS between December 2012 and December 2017. These time intervals were chosen because all the required atmospheric data were available and collected on a retrospective basis. We defined premature ACS as coronary disease diagnosed before 55 years of age [5]. Patients with one of two forms of ACS, either ST-elevation myocardial infarction (STEMI) or non-ST-elevation ACS (NSTE-ACS), were included in this study. STEMI was diagnosed as symptoms characteristic of cardiac ischemia with persistent ST-segment elevation on electrocardiography. Patients with NSTE-ACS included those with non-ST-elevation myocardial infarction (NSTEMI) or unstable angina. NSTEMI was diagnosed based on the presence of persistent ischemic symptoms with elevated cardiac troponin levels but no ST-segment elevation on electrocardiography. Unstable angina produced symptoms suggestive of cardiac ischemia without elevated cardiac troponin levels. A number of clinical variables were prospectively recorded: coronary risk factors, age and gender, previous vascular disease, the presence of asthma or chronic obstructive pulmonary disease, chronic kidney disease, atrial fibrillation, the number of affected vessels (as determined via coronary angiography), left ventricular ejection fraction, Killip class, troponin I concentration, coronary interventions (percutaneous transluminal coronary angioplasty or coronary revascularization surgery), duration of hospital stay, in-hospital mortality, and mortality at one year. The study was approved by the Clinical Research Ethics Committee of a tertiary hospital.

### 2.2. Air Quality and Meteorological Variables

Each day, we prospectively recorded the climatic data, with the concentrations of PM_10_, PM_2.5–10_ PM_2.5_, and gaseous pollutants (sulfur dioxide, nitrogen dioxide, and tropospheric ozone) between December 2012 and December 2017. These data were obtained from the Air Quality Network of the Canary Islands, which forms part of the European Air Quality Network and monitors the presence of particulate (PM_10_ and PM_2.5_) and gaseous pollutants, based on the mandatory reference methods and procedures established by the European Union (Directive 2008/50/EC). With an hourly resolution, the network produces the data that are transmitted to the European Environment Agency. The mean daily values were determined and analyzed. The occurrence of desert dust episodes was detected with the validated predictions of the *Sand and Dust Storm—Warning Advisory and Assessment System* (WMO SDS-WAS) of the World Meteorological Organization, with the Spanish State Meteorological Agency and Barcelona Supercomputing Center acting as regional reference centers for northern Africa, the Middle East, and Europe. The WMO SDS-WAS dust-prediction system consists of a meteorological prediction model that also includes a module for dust simulation (cycle of emissions, transport, and deposit) [7].

### 2.3. Statistical Analysis

Quantitative and categorical variables were reported as the mean ± standard deviation (SD) and number (%), respectively. Comparisons of the PM levels according to the number of ACS events per day were made using an analysis of variance (ANOVA).

Use was made of time series regression analyses, as these are commonly employed to study associations between pooled exposures and health outcomes. The result is a count (generally the number of events per day), and the main unit of analysis is the day, not the individual person. Since the individual confounding factors (e.g., age, gender, diabetes) do not vary significantly from one day to another, these factors have no impact on the effect estimates of the environmental variables. To control for seasonality and long-term trends, we used a case-crossover time-stratified design, with the strata being the day of the week within the same month [8,9,10]. In other words, the number of events in a day of the week, such as, for example, Tuesday, were compared with the number of events in the rest of Tuesdays of that same month. In this way, we controlled for the patient baseline characteristics and changes occurring over time, such as the seasons, demographic changes, day of the week, or any time trend.

Conditional logistic regression is used in many studies that analyze the impact of environmental variables on clinical events. However, due to the influence of non-measured causes, the counts of observed events usually show variations wider than those predicted by a Poisson distribution [8,9,10]. When a logistic regression model is used, such overdispersion cannot be controlled, because the results are binary and thus overdispersion, although present, is not apparent. However, the model implicitly assumes that there is no excessive dispersion of the counts [8,9,10]. Furthermore, conditional logistic regression assumes that the observations are independent. However, observations that lie close in time are probably more similar than those that are distant in time [8,9,10], and autocorrelation will very probably be present. The logistic regression model cannot be adjusted for overdispersion and autocorrelation—both being frequent causes of underestimated uncertainty and type 1 error.

To overcome these methodological limitations and based on the method of Armstrong et al. [9], we used a conditional Poisson regression model capable of controlling for overdispersion and autocorrelation. The dependent variable was the number of ACS events diagnosed per day. For this analysis, we had to modify the data matrix (Figure S1 of the Supplementary Materials).

The incidence rate ratio (IRR) was calculated with its 95% confidence interval (CI). In order to determine the influence of PM_10_, an adjustment was made for the following pollutants and environmental variables: PM_2.5–10_, PM_2.5_, sulfur dioxide, nitrogen dioxide, tropospheric ozone, temperature, and humidity. The maximum model was constructed with PM_10_, the aforementioned pollutants, environmental variables, and two-way interactions between PM_10_ and the rest of the variables. These interactions were evaluated and eliminated if no statistical significance was observed. We also took into account the possibility that the impact of PM could be produced some days before the occurrence of ACS. Therefore, we studied the desert dust intrusion on days 0 to 5 before the occurrence of ACS.

We also adjusted these lag effects for each other effect (e.g., the effect referred to PM_10_ of one lag day controlling for the effect of 2 lag days), developing a stratified distribution model [8,9,10]. However, in this latter model, it is probable that the effects in the different lag days are closely correlated. In order to reduce collinearity of the lag days, we placed some restrictions on the model; we postulated that the influence of PM_10_ for lag days 0, 1, and 2 and also for days 3, 4, and 5 is the same, in line with the recommendations of Armstrong et al. [9]. Lastly, the diagnoses of the model were assessed. Statistical significance was considered for *p* < 0.05. All the statistical analyses were carried out using the STATA version 16 package (StataCorp, College Station, TX, USA).

## 3. Results

### 3.1. Study Population

A total of 2416 patients were diagnosed with ACS during the study inclusion period. There were 649 women (26.9%), and the mean age was 63.9 ± 12.5 years. The in-hospital mortality rate was 5.3% (n = 129). A total of 1730 patients were over 55 years of age and 686 were under 55 years of age. The characteristics of the patients under and over 55 years of age are shown in Table 1. The proportion of women in the under 55 years of age ACS group was significantly lower than that in the over 55 years of age ACS group. Furthermore, patients under 55 years of age with ACS had a lower frequency of arterial hypertension, dyslipidemia, diabetes mellitus, chronic obstructive pulmonary disease, chronic kidney disease, and previously known coronary disease. More multivessel lesions were observed in the over 55 years of age ACS group. The in-hospital mortality was lower in the under 55 years of age group than in the over 55 years of age ACS group.

### 3.2. Patients with ACS under 55 Years of Age

During the 1824 days of the study, there was one admission due to ACS in a patient under 55 years of age at 481 days, two admissions due to ACS at 83 days, and three admissions due to ACS at 13 days. The global incidence of ACS in patients under 55 years was 0.38 ACS per day. Table 2 reports the PM_10_ concentrations according to the number of admissions. The impact of PM_10_ in the air on the risk of suffering ACS on the same day was (per 10 μg/m^3^) IRR = 1.1 (95% CI 0.8–1.52; *p* = 0.55). Table 3 shows the impact of PM, gaseous pollutants, temperature, and humidity according to the Poisson regression model.

**Figure 1:**
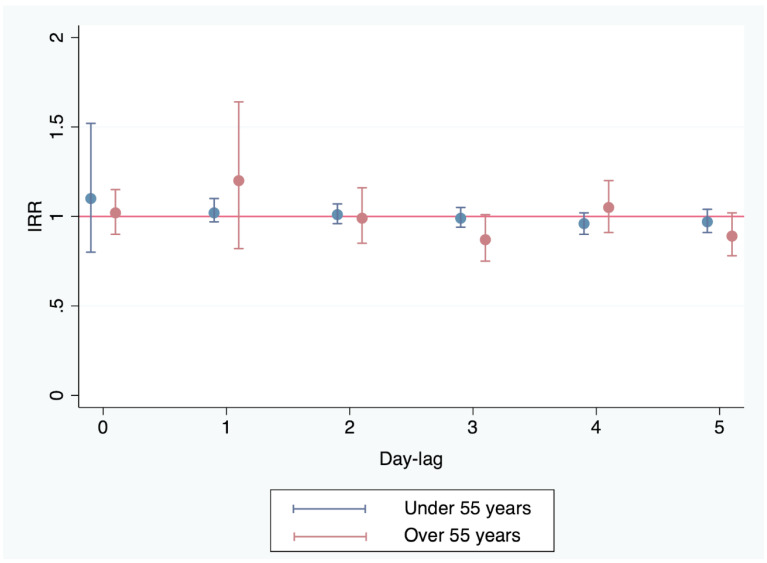
Impact of the PM_10_ concentrations in the air on the incidence of ACS in patients under and over 55 years of age. The influence of these concentrations in the previous 5 days are reported. IRR: incidence rate ratio.

No significant impact of PM_10_ was observed for any of the studied days prior to the onset of ACS. Specifically, on the day before the syndrome, IRR = 1.02 (95% CI 0.97–1.1; *p* = 0.41); two days before, IRR = 1.01 (95% CI 0.96–1.07; *p* = 0.66); three days before, IRR = 0.99 (95% CI 0.94–1.05; *p* = 0.78); four days before, IRR = 0.96 (95% CI 0.9–1.02; *p* = 0.18); and five days before, IRR = 0.97 (95% CI 0.91–1.04; *p* = 0.41). Figure 1 shows the IRR values with the corresponding confidence intervals.

**Figure 2:**
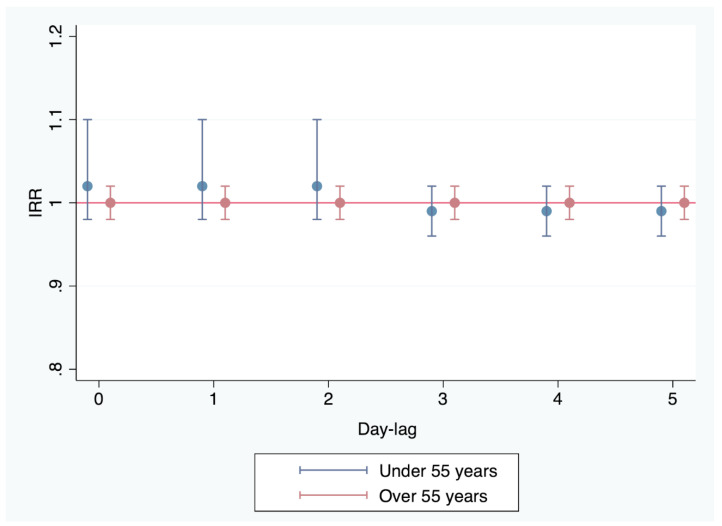
Impact of the PM_10_ concentrations in the air on the incidence of ACS in patients under and over 55 years of age, controlled for different lag effects with restrictions (to avoid collinearity), assuming that the influence is the same for the previous 0, 1, and 2 days and for the previous 3, 4, and 5 days. IRR: incidence rate ratio.

No association was found in the model with restrictions assuming that the effect on day 0 and days 1 and 2 before ACS presented the same IRR = 1.02 (95% CI 0.98–1.1; *p* = 0.38); the same applied to days 3, 4, and 5 before, with IRR = 0.99 (95% CI 0.96–1.02; *p* = 0.51). Figure 2 shows the IRR values with the corresponding confidence intervals.

### 3.3. Patients with ACS over 55 Years of Age

During the 1824 days of the study, there was one admission due to ACS in a patient over 55 years of age at 646 days, two admissions at 321 days, three admissions at 98 days, four admissions at 32 days, and five admissions at 4 days. The global incidence of ACS in patients over 55 years was 0.95 ACS per day. Table 4 reports the PM_10_ concentrations according to the number of admissions. The impact of PM_10_ in the air on the risk of suffering ACS on the same day was (per 10 μg/m^3^) IRR = 1.02 (95% CI 0.9–1.15; *p* = 0.74).

No significant impact of PM_10_ was observed for any of the studied days prior to the onset of ACS. Specifically, on the day before the syndrome, IRR = 1.2 (95% CI 0.82–1.64; *p* = 0.38); two days before, IRR = 0.99 (95% CI 0.85–1.16; *p* = 0.91); three days before, IRR = 0.87 (95% CI 0.75–1.01; *p* = 0.055); four days before, IRR = 1.05 (95% CI 0.91–1.2; *p* = 0.49); and five days before, IRR = 0.89 (95% CI 0.78–1.02; *p* = 0.09). Figure 1 shows the IRR values with the corresponding confidence intervals (CIs).

No association was found in the model with restrictions assuming that the effect on day 0 and days 1 and 2 before ACS presented the same IRR = 1 (95% CI 0.98–1.02; *p* = 0.69); the same applied to days 3, 4, and 5 before, with IRR = 1 (95% CI 0.98–1.02). Figure 2 shows the IRR values with the corresponding confidence intervals.

## 4. Discussion

The present study analyzed the possibility that desert dust produced by natural phenomena can trigger ACS in subjects of a younger age. The results obtained indicate that exposure to Saharan dust is not a potential triggering cause of ACS in subjects younger than 55 years of age.

Climate change and the global air quality crisis are having an impact on numerous aspects of society [1]. The concentrations of pollutants that affect air quality can vary markedly in a matter of a few days, depending on the daily emissions and particularly on the meteorological conditions. Due to variations in wind speed, the vertical stability of the atmosphere, or the arrival of polluted air from other regions, the concentrations of these pollutants may vary between 50 and 500% in just a few days [11].

The proximity of the Canary Islands to the African continent defines the islands as an area of great interest, taking into account the frequency of intrusions of desert dust mineral components and their impact on the population. It is therefore very important to characterize the intrusions of desert dust that affect the archipelago [2]. Sand storms are common in desert regions and lift an amalgam of fine polluting particles into the air. These particles, if inhaled, can have a negative impact on cardiovascular health. These particles transported with the wind may contain chemical compounds and heavy metals that trigger a series of inflammatory responses after penetrating into the body [3,12].

Studies on the influence of desert dust on ACS are few, and the results are moreover discordant. Three recent studies have examined the relationship between dust storms and ACS [6,13,14]. A recent study in Spain found no association between PM_10_ concentrations derived from desert dust and the incidence of ACS [6]. Kojima et al. [13] studied the effect of desert dust on the incidence of myocardial infarction and concluded that desert dust episodes the day before lead to the appearance of ACS (odds ratio [OR] = 1.46; 95% CI 1.09–1.95). Vodonos et al. [14] reported an increased incidence of ACS related to PM_10_ (with one lag day) during the days of a dust storm (OR = 1.007; 95% CI 1.002–1.012), though no significant impact was observed on the days without this atmospheric phenomenon (OR = 1.011; 95% CI 0.998–1.025). It therefore may be inferred that cardiovascular pathophysiology may differ in terms of the impact of PM_10_ generated by combustion processes and those of a natural origin (desert dust), suggesting likely variations in their influence [1,12,15,16].

While the precise biological mechanisms linking air pollution and ACS remain incompletely understood, hypotheses abound [17]. Controlled human exposure and animal experiments suggest that short-term particulate exposure may heighten thrombogenicity via platelet activation, oxidative stress, and cytokine interactions [17,18]. Additionally, endothelial dysfunction, inflammation, dyslipidemia, and vascular dysregulation are implicated pathways [18]. Moreover, atmospheric aerosol PM can also contain microplastics and nanoplastics [19]. Recently, Marfella et al. demonstrated that micro and nanoplastics can have direct impacts on human health [20].

Due to the use of ambiguous language in reference to desert dust episodes, the uncertainty regarding the composition of the particles during such episodes, and the presence of undocumented or uncontrolled factors, the epidemiological relationships between desert dust and ACS generate significant controversy, with a lack of firm conclusions [6]. The present study evaluated desert dust events according to the PM_10_ concentrations, applying a control to mitigate possible confounding factors (particularly other particles in suspension and pollutants), and a novel and appropriate statistical method was used to avoid underestimating type 1 errors [8,9,10]. In addition, our study was conducted before COVID-19, so patients were not wearing surgical masks.

## 5. Limitations

Our study has certain limitations. Firstly, the measurements were made at static points to assess the atmospheric pollutant concentrations in the exterior, despite the fact that people spend most of their time indoors. Secondly, the challenge of correctly measuring exposure is a basic limitation in studies comprising a time series, since the average taken from static monitoring stations does not precisely represent the mean exposure experienced by the population. This could result in downward bias in the estimations of time-series analyses, as suggested by some data [21]. Given that the effect size of dust particles on the incidence of ACS may be relatively small [15], a larger sample size than that in the present study may have detected a significant association, but the clinical implications of such a minor contribution would be uncertain. Moreover, our study had a retrospective design. We are well aware that prospective longitudinal studies are suitable for these studies. Lastly, the inconsistencies in the existing evidence suggest that more in-depth studies of the characteristics associated with the short-term effects of desert dust are needed, with special attention on its composition and biological properties [22].

## 6. Conclusions

This pioneering study analyzes the possible links between desert dust episodes and ACS in subjects of a younger age. The findings suggest that desert dust is unlikely to be related to the incidence of ACS in subjects younger than 55 years of age.

## Figures and Tables

**Table 1:** Clinical and hemodynamic characteristics of the study population.

Variable	Under 55 Years N = 686	Over 55 Years N = 1730	*p*-Value
Age (years)	48.9 ± 5	69.9 ± 9	<0.001
Women	131 (19.1%)	518 (29.9%)	<0.001
Arterial hypertension	285 (41.6%)	1257 (72.7%)	<0.001
Dyslipidemia	333 (48.6%)	1067 (61.7%)	<0.001
Diabetes	129 (18.8%)	754 (43.6%)	<0.001
Asthma	24 (3.5%)	58 (3.4%)	0.9
Chronic obstructive pulmonary disease	10 (1.5%)	122 (7.1%)	<0.001
Chronic kidney disease	12 (1.8%)	243 (14.1%)	<0.001
Previously known coronary disease	108 (15.8%)	566 (32.7%)	<0.001
Supraventricular arrhythmia	3 (0.4%)	146 (8.4%)	<0.001
Acute myocardial infarction	438 (63.9%)	912 (52.8%)	<0.001
Left ventricular dysfunction	124 (18.1%)	439 (25.4%)	<0.001
Killip class			<0.001
I	648 (94.5%)	1423 (82.3%)	
II	12 (1.8%)	146 (8.5%)	
III	2 (0.3%)	46 (2.7%)	
IV	24 (3.5%)	113 (6.5%)	
Coronary artery lesions			<0.001
No lesions	43 (6.4%)	135 (8.4%)	
Single-vessel disease	409 (60.5%)	639 (39.7%)	
Two-vessel disease	158 (23.4%)	442 (27.5%)	
Three-vessel disease	66 (9.8%)	394 (24.5%)	
Troponin I peak (ng/mL)	26.6 ± 29.7	21.2 ± 27.7	<0.001
Percutaneous transluminal coronary angioplasty treatment	552 (80.5%)	1090 (63.1%)	<0.001
Surgical treatment	33 (4.8%)	137 (7.9%)	0.01
In-hospital mortality	9 (1.3%)	120 (6.9%)	<0.001
One-year mortality among the survivors	3 (0.4%)	15 (0.9%)	0.43

**Table 2:** PM_10_ concentrations (µg/m^3^) according to the number of ACS events per day in patients under 55 years of age.

Day	No ACS/Day N = 1246	1 ACS/Day N = 481	2 ACS/Day N = 83	3 ACS/Day N = 13	*p*-Value
Day of admission PM_10_ (μg/m^3^)	21.5 ± 23.4	21.5 ± 22.2	20.6 ± 18.7	14.3 ± 7.8	0.71
1 Day before PM_10_ (μg/m^3^)	21.3 ± 21.2	21.4 ± 25.8	24 ± 28.1	16.2 ± 10.7	0.62
2 Days before PM_10_ (μg/m^3^)	20.8 ± 19.2	22.6 ± 29.3	24.4 ± 29.2	16.6 ± 11.3	0.24
3 Days before PM_10_ (μg/m^3^)	21.3 ± 21.6	21.7 ± 26.9	20.4 ± 13.2	17.2 ± 13.4	0.87
4 Days before PM_10_ (μg/m^3^)	21.7 ± 24.2	20.6 ± 20.3	19.8 ± 12.9	20.8 ± 14.4	0.75
5 Days before PM_10_ (μg/m^3^)	21.6 ± 23.8	20.8 ± 21.2	20.6 ± 16.1	19.2 ± 15	0.89

ACS: Acute coronary syndrome; PM: particulate material; PM_10_: particulate material with a diameter <10 µm. N indicates the number of days during the study period.

**Table 3:** Influence of the levels of PM, gaseous pollutants, temperature, and humidity on the incidence of ACS (on the day of admission) in patients under 55 years of age. The influence of PM is reported per variations of 10 μg/m^3^.

Atmospheric Variable	IRR (95% CI)
PM_10_ (μg/m^3^)	1.1 (95% CI 0.8–1.52)
PM_2.5–10_ (μg/m^3^)	0.84 (95% CI 0.55–1.29)
PM_2.5_ (μg/m^3^)	1.01 (95% CI 0.82–1.24)
SO_2_ (μg/m^3^)	1.01 (95% CI 0.91–1.1)
NO_2_ (μg/m^3^)	1.03 (95% CI 0.98–1.1)
O_3_ (μg/m^3^)	1 (95% CI 0.99–1.01)
Temperature (°C)	1 (95% CI 0.89–1.1)
Humidity (%)	1 (95% CI 0.99–1)

PM: particulate material; PM_10_: particulate material with a diameter <10 μm; PM_2.5–10_: particulate material with a diameter 2.5–10 μm; PM_2.5_: particulate material with a diameter <2.5 μm; SO_2_: sulfur dioxide; NO_2_: nitrogen dioxide; O_3_: tropospheric ozone.IRR: incidence rate ratio.

**Table 4:** PM_10_ concentrations (µg/m^3^) according to the number of ACS events per day in patients over 55 years of age.

Day	No ACS/Day N = 723	1 ACS/Day N = 646	2 ACS/Day N = 321	3 or More ACS/Day N = 134	*p*-Value
Day of admission PM_10_ (μg/m^3^)	22.1 ± 25.9	20.4 ± 19.3	22.2 ± 24.2	20.4 ± 15.5	0.84
1 Day before PM_10_ (μg/m^3^)	22.5 ± 25.8	21.1 ± 22.7	20.9 ± 14.7	18.1 ± 21.12	0.64
2 Days before PM_10_ (μg/m^3^)	22 ± 22.8	20.8 ± 22.4	21.4 ± 23.9	21.8 ± 23.4	0.89
3 Days before PM_10_ (μg/m^3^)	21.9 ± 22.4	20.5 ± 21.6	22 ± 26.4	20.7 ± 23.1	0.85
4 Days before PM_10_ (μg/m^3^)	21.1 ± 20.9	21.3 ± 22.4	22 ± 26.8	21.1 ± 25.7	0.99
5 Days before PM_10_ (μg/m^3^)	21.4 ± 24.4	20.5 ± 19.5	22.2 ± 24.9	23.9 ± 25.9	0.75

ACS: Acute coronary syndrome; PM: particulate material; PM_10_: particulate material with a diameter <10 µm. N indicates the number of days during the study period.

## Data Availability

The data presented in this study are available on request from the corresponding author.

## Supplementary Materials

The following supporting information can be downloaded at: https://www.mdpi.com/article/10.3390/jcm13082392/s1, Figure S1: Data matrix for Poisson regression.

## References

[1] Benito Lozano M., Rodríguez S. (2023). La imperiosa necesidad de programar la asistencia a urgencias, atendiendo al cambio climático y las crisis de calidad del aire. Rev. Esp. Urg. Emerg..

[2] Rodríguez S., Cuevas E., Prospero J.M., Alastuey X., Querol J., López-Solano M., García I., Alonso-Pérez S. (2015). Modulation of Saharan dust export by the North African dipole.. Atmos. Chem. Phys..

[3] Dominguez-Rodriguez A., Rodríguez S., Baez-Ferrer N., Abreu-Gonzalez P., Abreu-Gonzalez J., Avanzas P., Carnero M., Moris C., López-Darias J., Hernández-Vaquero D. (2020). Impact of Saharan dust exposure on airway inflammation in patients with ischemic heart disease. Transl. Res..

[4] Domínguez-Rodríguez A., Báez-Ferrer N., Abreu-González P., Rodríguez S., Díaz R., Avanzas P., Hernández-Vaquero D. (2021). Impact of Desert Dust Events on the Cardiovascular Disease: A Systematic Review and Meta-Analysis. J. Clin. Med..

[5] Liu J.D., Gong R., Xu J.S., Zhang S.Y., Wu Y.Q. (2023). Clinical Characteristics and Outcomes of Chinese Patients with Premature Acute Coronary Syndrome. Int. Heart J..

[6] Domínguez-Rodríguez A., Rodríguez S., Baez-Ferrer N., Avanzas P., Abreu-González P., Silva J., Morís C., Hernández-Vaquero D. (2021). Impact of Saharan dust on the incidence of acute coronary syndrome. Rev. Esp. Cardiol..

[7] Pérez C., Haustein K., Janjic Z., Jorba O., Huneeus N., Baldasano J.M., Black T., Basart S., Nickovic S., Miller R.L. (2011). Atmospheric dust modeling from meso to global scales with the online NMMB/BSC-Dust model—Part 1: Model description, annual simulations and evaluation. Atmos. Chem. Phys..

[8] Lu Y., Zeger S.L. (2007). On the equivalence of case-crossover and time series methods in environmental epidemiology. Biostatistics.

[9] Armstrong B.G., Gasparrini A., Tobias A. (2014). Conditional Poisson models: A flexible alternative to conditional logistic case cross-over analysis. BMC Med. Res. Methodol..

[10] Bhaskaran K., Gasparrini A., Hajat S., Smeeth L., Armstrong B. (2013). Time series regression studies in environmental epidemiology. Int. J. Epidemiol..

[11] Bañeras J., Iglesies-Grau J., Téllez-Plaza M., Arrarte V., Báez-Ferrer N., Benito B., Campuzano Ruiz R., Cecconi A., Domínguez-Rodríguez A., Rodríguez-Sinovas A. (2022). Environment and cardiovascular health: Causes, consequences and opportunities in prevention and treatment. Rev. Esp. Cardiol..

[12] Münzel T., Hahad O., Sørensen M., Lelieveld J., Duerr G.D., Nieuwenhuijsen M., Daiber A. (2022). Environmental risk factors and cardiovascular diseases: A comprehensive expert review. Cardiovasc. Res..

[13] Kojima S., Michikawa T., Ueda K., Ueda K., Sakamoto T., Matsui K., Kojima T., Tsujita K., Ogawa H., Nitta H. (2017). Asian dust exposure triggers acute myocardial infarction. Eur. Heart J..

[14] Vodonos A., Friger M., Katra I., Krasnov H., Zahger D., Schwartz J., Novack V. (2015). Individual Effect Modifiers of Dust Exposure Effect on Cardiovascular Morbidity. PLoS ONE.

[15] de Longueville F., Ozer P., Doumbia S., Henry S. (2013). Desert dust impacts on human health: An alarming worldwide reality and a need for studies in West Africa. Int. J. Biometeorol..

[16] Bañeras J., Ferreira-González I., Marsal J.R., Barrabés J.A., Ribera A., Lidón R.M., Domingo E., Martí G., García-Dorado D. (2018). Short-term exposure to air pollutants increases the risk of ST elevation myocardial infarction and of infarct-related ventricular arrhythmias and mortality. Int. J. Cardiol..

[17] Robertson S., Miller M.R. (2018). Ambient air pollution and thrombosis. Part Fibre Toxicol..

[18] Chen R., Jiang Y., Hu J., Chen H., Li H., Meng X., Ji J., Gao Y., Wang W., Liu C. (2022). Hourly Air Pollutants and Acute Coronary Syndrome Onset in 1.29 Million Patients. Circulation.

[19] Sridharan S., Kumar M., Singh L., Bolan N.S., Saha M. (2021). Microplastics as an emerging source of particulate air pollution: A critical review. J. Hazard Mater..

[20] Marfella R., Prattichizzo F., Sardu C., Fulgenzi G., Graciotti L., Spadoni T., D’Onofrio N., Scisciola L., La Grotta R., Frigé C. (2024). Microplastics and Nanoplastics in Atheromas and Cardiovascular Events. N. Engl. J. Med..

[21] Zeger S.L., Thomas D., Dominici F., Samet J.M., Schwartz J., Dockery D., Cohen A. (2000). Exposure measurement error in time-series studies of air pollution: Concepts and consequences. Environ. Health Perspect..

[22] Domínguez-Rodríguez A., Avanzas P., Báez-Ferrer N., Abreu-González P., Rodríguez S., Matos-Castro S., Hernández-Vaquero D. (2023). Rationale and Design of the Impact of Air Pollution due to DESERT Dust in Patients with HEART Failure (DESERT HEART). J. Clin. Med..

