# Supplementary figures and images for: The Association of Desert Dust with the Risk of Acute Coronary Syndrome in Subjects of a Younger Age
Source: J Clin Med. 2024 Apr 19;13(8):2392. doi: 10.3390/jcm13082392 (PMC11051357; doi:10.3390/jcm13082392)

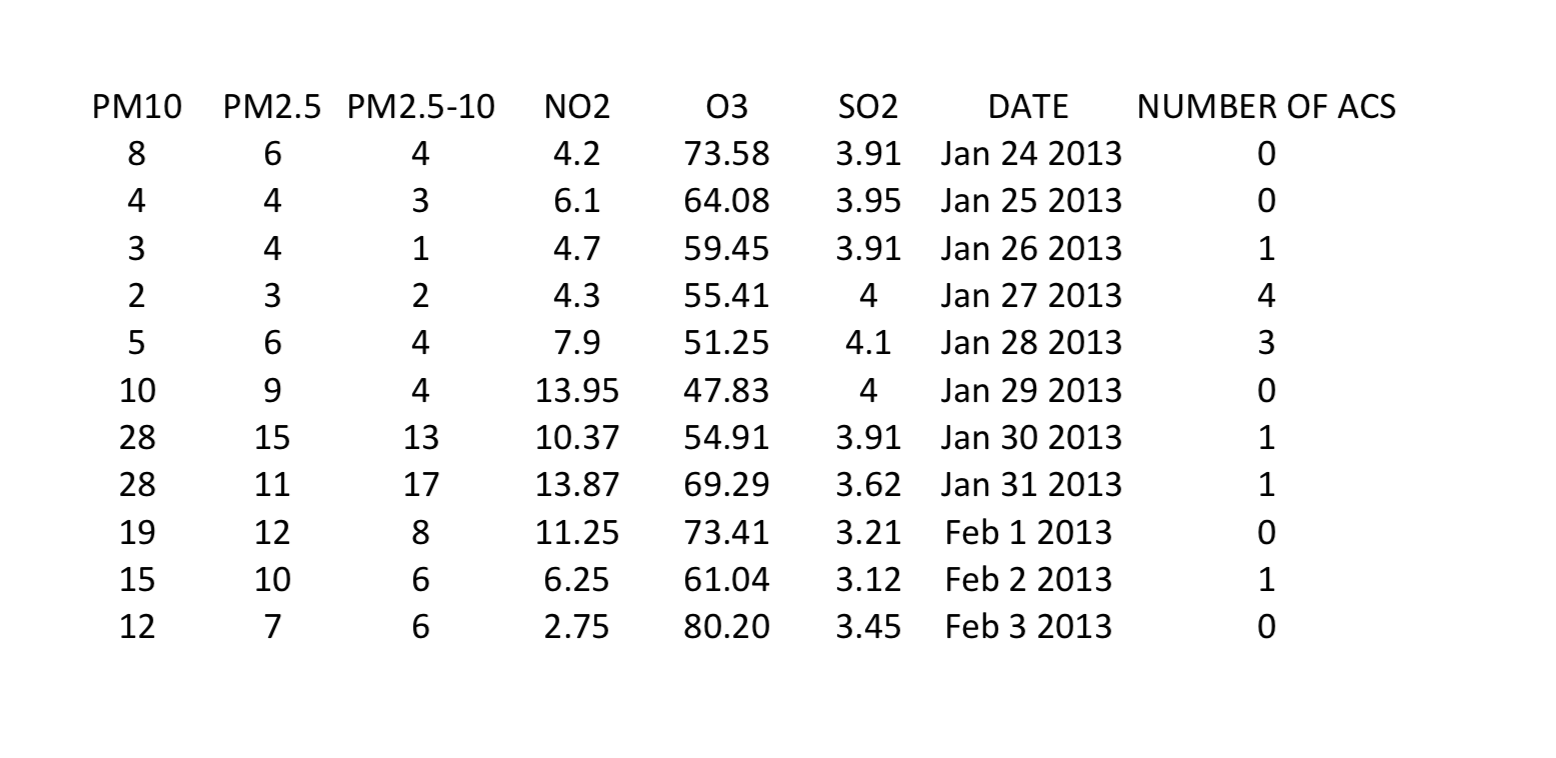

Supplement: Supplementary file 1 [file jcm-13-02392-s001.zip › Figure S1.tiff]
